# Lung Transplantation for Pulmonary Vascular Disease in Children: A United Network for Organ Sharing Analysis

**DOI:** 10.21203/rs.3.rs-3310701/v1

**Published:** 2023-09-06

**Authors:** Hosam F. Ahmed, Amalia Guzman-Gomez, Malika Desai, Alia Dani, David Morales, Paul J. Critser, Farhan Zafar, Don Hayes

**Affiliations:** Cincinnati Children’s Hospital Medical Center; Cincinnati Children’s Hospital Medical Center; Cincinnati Children’s Hospital Medical Center; Cincinnati Children’s Hospital Medical Center; Cincinnati Children’s Hospital Medical Center; Cincinnati Children’s Hospital Medical Center; Cincinnati Children’s Hospital Medical Center; Cincinnati Children’s Hospital Medical Center

**Keywords:** Congenital heart disease, Lung transplant, Pulmonary vascular disease

## Abstract

**Background:**

Pulmonary vascular disease (PVD) represents an important clinical indication for lung transplant (LTx) in infants, children, and adolescents. There is limited information on LTx outcomes in these patients. We explored LTx volumes and post-LTx survival in children with PVD compared to other diagnoses.

**Methods:**

The UNOS Registry was queried from 1989–2020 to identify first-time pediatric LTx recipients (<18 yo). PVD was categorized as idiopathic pulmonary arterial hypertension (IPAH) and non-idiopathic arterial hypertension (non-IPAH) and compared to all other patients as other diagnoses. Univariate and multivariate regression models were performed.

**Results:**

984 pediatric LTx patients (593 before 2010 and 391 during/after 2010) were identified, of which 145 (14.7%) had PVD. There has been no significant change in annual rate of all LTxs over comparative eras. However, there has been a decrease in rate of LTxs for PVD patients. Children with PVD had similar survival to other LTx groups in the early era (p=0.2) and the latter era (p=0.9). Univariate Cox models, showed that LTx in patients with PVD was associated with a significantly less risk of mortality for children aged 6–11 years compared to younger and older cohorts (HR=0.4 [0.17–0.98];p=0.045), whereas multivariate analysis showed a trend towards higher mortality in 11–17-year-olds (HR=1.54 [0.97–2.45];p=0.06). For PVD patients, oxygen supplementation and ventilator support at LTx were associated with worse post-transplant survival (p=0.029 and p=0.01).

**Conclusions:**

There has been a decrease in LTx volume for pediatric patients with PVD in the modern era. Post-LTx outcomes for children with PVD are similar to those of other diagnoses in both eras, with children aged 6–11 years having the best survival. Given these findings, LTx should be considered for this patient population.

## Introduction

Pulmonary vascular disease (PVD) has been a common indication for lung trasnplant (LTx) in children and adults [1–3]. In the early era of LTx, there were no targeted pharmacotherapeutic options for pulmonary hypertension (PH). Since 2000, there has been an increasing number of PH therapies introduced and used for groups of children with PVD [4]. The majority of these therapies were initially and remain approved only in adults by the United States (US) Food and Drug Administration. Nonetheless, the use of these medications in children with certain categories of PVD has been expanded gradually in recent years [5]. Treatment with these pharmacotherapies in children with PVD offer a treatment option that may reduce or avoid the need for LTx. At the same time, hospitalizations and associated morbidity of pediatric patients with PVD have increased significantly during the past two decades [6]. These patients represent a morbid population with a high rate of rehospitalization, need for intensive care unit (ICU) care, and mortality [7].

In the face of this changing clinical landscape for children with PH, we explored trends in pediatric LTx in this population over time and assessed current outcomes for these high risk patients. Whereas the adult LTx population has experienced a doubling in the number of worldwide LTxs performed since 2004, the number of pediatric LTxs has not and decreased in some pulmonary conditions outside of PVD [1–3, 8, 9]. Historically, cystic fibrosis (CF) has been the most common indication for pediatric LTx, but there has been a downward trajectory in the number of LTxs performed in children with a slightly upward trajectory in the number performed in children with PVD, including idiopathic pulmonary arterial hypertension (IPAH) and non-IPAH [2, 4–8].

Most published reports investigating post-LTx outcomes for children with PVD have been limited to single-center studies, examining outcomes primarily in patients with IPAH [10–12]. The sole multi-institutional study on this subject had a small sample size of only 23 children[13]. Recently, an analysis of the United Network for Organ Sharing (UNOS) Registry evaluated post-transplant outcomes of 65 children with IPAH who underwent thoracic transplant (47 LTxs, 18 heart-lung transplants) over a 10-year period [14]. In that analysis, the investigators showed 5-year survival rates of 61% for LTx and 48% for heart-lung transplant, with post-transplant outcomes being similar to children transplanted for other indications [14]. Due to the changing epidemiology for LTx in children with PVD, the introduction of targeted PH pharmacotherapies, and a gap in the medical literature on PVD in children, we sought to to assess current post-LTx outcomes in the pediatric population using a publicly accessible database in the US.

## Methods

### Data Collection

**Figure 1:**
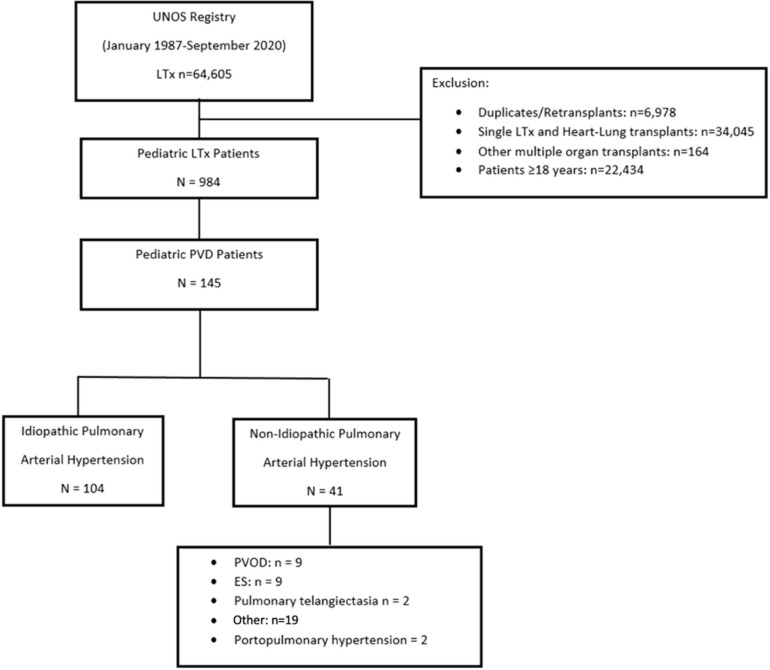
Flowchart for cohort selection

We retrospectively evaluated data from LTx recipients who were registered in the Organ Procurement and Transplant Network (OPTN) Standard Analysis and Research Database administered by UNOS since 1987 [15]. This study was approved by the local Institutional Review Board at Cincinnati Chldren’s Hospital Medical Center with a waiver of the need for individual consent. The UNOS/OPTN database was queried for all patients under 18 years of age who underwent primary bilateral LTx from January 1990 to September 2020 and were, diagnosed with PVD. Patients with PVD included those with IPAH and those with non-IPAH. All remaining patients who did not classify as PVD were categorized as other diagnoses. Patients were excluded if their LTx was re-transplantation, or if they received a multiple organ transplant, single LTx, and had unknown survival post-LTx (Fig. 1). The era of LTx was defined as before (<2010, termed early era) and afterwards 2010 (≥ 2010, termed modern era).

### Covariates

The main covariates included human leukocyte antigen (HLA) mismatch, mean pulmonary artery pressure (mPAP) (mmHg), estimated glomerular filtration rate (eGFR) (mL/min/1.73m^2^), bilirubin level (mg/dL), age, supplemental oxygen requirement (O_2_; liters per minute (LPM)), ventilator support, and donor cause of death at time of LTx. Pulmonary function was analyzed through forced vital capacity percent predicted (FVC%) and forced expiratory volume in one second percent predicted (FEV1%). Ischemic time for the transplant was measured in hours. Sex and race/ethnicity of both the donor and recipient were included in our analysis.

### Statistical Methods

All analyses were performed using SPSS (IBM Corp. Released 2020. IMB SPSS Statistics, Version 27.0. Armon, NY: IMB Corp). Baseline characteristics were determined using medians with interquartile range [IQR] for continuous measures, and frequency with percentages for categorical variables. LTx frequency difference between PVD patients and other patients was obtained using paired sample test for mean comparison. For analyses, p-value < 0.05 was considered statistically significant. Comparisons between groups were done using Student’s t-test for continuous variables & Pearson’s chi-squared test with Yates’ continuity correction for discrete variables.. The primary outcome of the study was to compare post transplant long term survival. Kaplan-Meier surival models were created for comparison of survival between patients who underwnt LTx before or after 2010. Univariable cox regression analysis was done to identify risk factors for mortality among PVD patients underwent LTx. Multivariable regression analysis was conducted including all pediatric LTx recipients. The cox proportional hazard model was adjusted for covariates.

## Results

A total of 984 children who underwent LTx were identified, of which 145 (14.7%) were diagnosed with PVD. Of the PVD patients, 104 (71.7%) had IPAH and 41 (28.3%) had non-IPAH. The details of cohort diagnoses are further described in Fig. 1. Of the entire LTx population, 59.3% (86/145) had PVD in the early era (< 2010) and 40.7% (59/145) in the modern era (≥ 2010) (p< 0.001). Comparing these two eras (early vs. modern), the proportion of pediatric LTxs performed for PVD were 8.7% and 5.9%, respectively.

Compared to other diagnoses, children with PVD were significantly younger (7 [1–14] vs 13 [8–16] years; p< 0.001) and had a higher percentage of non-White patients (52 (35.8%) vs 203 (24.2%); p = 0.003).Patients with PVD transplanted after 2005 (87 (607%)), after institution of the lung allocation score (LAS), had a lower LAS than patients with other diagnoses (33.4 [29.5–28.7] vs 36.3 [33.5–42.5]; p < 0.001). Additionally, children with PVD had a higher bilirubin level at transplant (0.5 [0.3–0.8] vs 0.3 [0.2–0.5] g/dL; p < 0.01) and a higher mPAP (67.5 [55.7–81.25] vs 28 [24–36] mmHg; p< 0.001) compared to those with other diagnoses. (Table 1). Children with PVD had higher pulmonary function, including FVC% and FEV1 %. Notably, children with PVD had a lower use of supplemental oxygen therapy at the time of LTx (Table 1).

**Figure 2:**
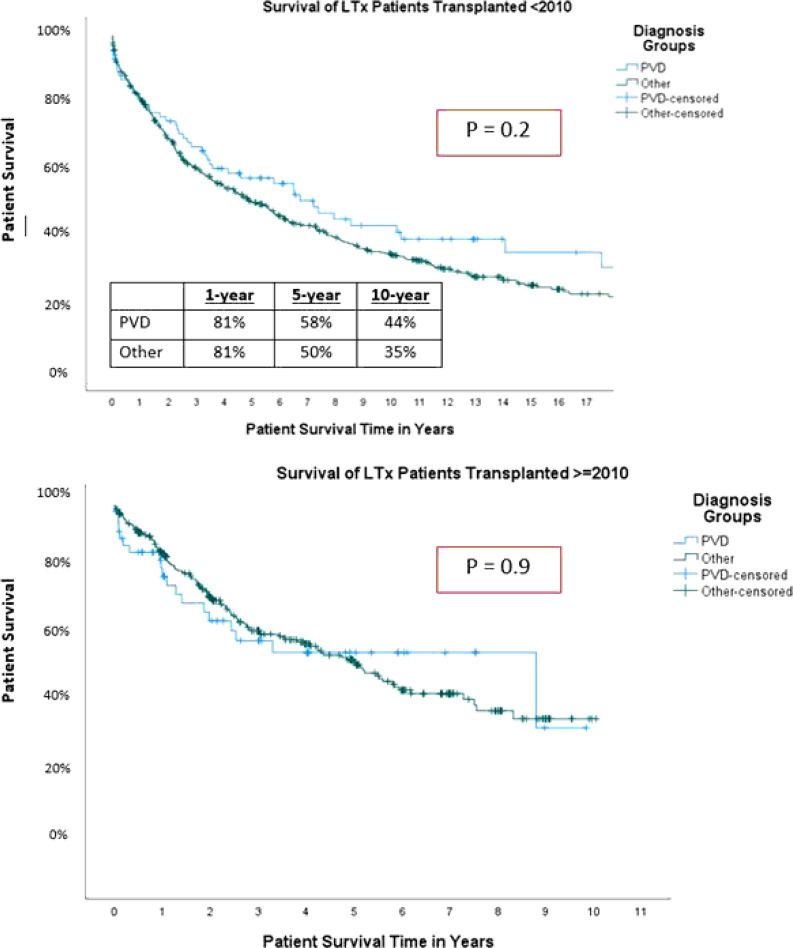
Survival analysis of pediatric lung transplant receipients in different eras. early era (<2010) and modern era (≥2010).

After transplantation, Kaplan-Meier analysis showed that at 1 year, 5-year and 10-year post transplant, patients with PVD had survival rates of 81 %, 58%, 44% and patients with other diagnoses had survival rates of 81 %, 50%, 35%, respectively, with no significant differences between the 2 groups in both the early and modern eras (p = 0.2 and p = 0.9, respectively) (Fig. 2). Upon univariable regression survival analysis, post-LTx mortality hazard in children with PVD was not significantly different between the early and modern eras (p = 0.88). With respect to recipient age, PVD recipients aged 6- to 11-years old demonstrated a significantly decreased mortality compared to other age cohorts. (HR = 0.4 [0.16–0.98]; p = 0.045). Furthermore, ventilatory support at time of LTx (HR = 2.05 [1.18–3.55]; p = 0.001), an ischemic time of more than 6 hours (HR = 1.86 [1.12–3.11]; p = 0.017), and supplemental oxygen level (HR = 1.07 [1.07–1.14]; p = 0.029) were associated with an increased mortality hazard for pediatric LTx recipients with PVD. Conversely, stroke as donor cause of death was protective (HR = 0.24 [0.07–0.83]; p = 0.024) (Table 2). Upon multivariable regression survival analysis of the whole cohort, none of the tested variables including PVD was found to be associated with post-transplant survival (Table 3).

## Discussion

Over the past two decades, there has been an increase in both the frequency and severity of PVD in children. Therefore, we conducted this study to examine the trends and effects of LTx in the pediatric population with PVD. It is concerning to note that despite the use of optimal pharmacotherapies, there has been a rise in the rates of morbidity and mortality among this group of patients. Surprisingly, the number of children undergoing LTx for PVD has decreased in recent times. This suggests that children with PVD are potentially missing the opportunity to benefit from LTx. Although a recent adult-focused consensus document outlined more suggestions on consideration of children with PVD as possible LTx candidates, specific guidance for the pediatric PVD population is not extensive and only focused on children with IPAH, and there is currently a lack of consensus in the medical literature regarding the referral and selection of this pediatric population for LTx [16].

While utilization of PH pharmacotherapies in children with PVD in lieu of LTx has risen [5], there is a highly variable success rate with these medications, and they are not curative for PVD. In the pediatric PVD population, especially when there is poor response to PH pharmacotherapies, there is significant morbidity and mortality requiring considerable use of healthcare resources in the US. Furthermore, patients with significantly high pulmonary vascular resistance that does not respond or minimally responds to optimal pharmacotherapy are at risk of experiencing persistent right heart failure, syncope, or even death. [17]. For instance, Goldstein and colleagues demonstrated that 25% of children with IPAH who were listed for transplant died while waiting, all developed suprasystemic right sided cardiac pressures [10]. Another study looked into post-transplant outcomes in children with IPAH and found that during the study period, there was a mortality rate of 40.7% amongst additional patients who were listed for but did not receive a LTx [12]. In addition to optimal medical therapy, non-transplant treatment options for children with progressing PVD include invasive procedures such as extracorporeal life support (ECLS) during episodes of acute deterioration and placement of a right-to-left shunt. ECLS can be used as a temporizing measure to bridge patients to LTx in selected patients. In some instances, redirecting blood flow from the pulmonary circulation to the systemic circulation through a right-to-left shunt can increase cardiac output. However, creating such a shunt can also lead to tissue perfusion issues due to the delivery of less oxygenated blood. The two most commonly used options for introducing a right-to-left shunt are creating an atrial communication or a reverse Potts shunt. For the past 30 years, in specific cases of severe PVD, an atrial septal communication has been successfully created using a blade and balloon in a catheterization laboratory. [18]. A recent single-center study, enrolling both adults and children, substantiated the benefit of the creation of atrial communication, with the majority of this study cohort having symptomatic right heart failure or pre-syncope/syncope. In this study, balloon atrial septostomy performed by experienced centers was found to be safe and effective. However, some patients may still require LTx even after the procedure, as they may remain quite ill. [19]. Although outcomes at experienced centers with reverse Potts shunt have been positive with improvement in patient functional status, the role of this procedure in patients with severe right ventricular dysfunction or significant lung disease remains unclear [20–23]. Additionally, long term outcomes for patients undergoing reverse Potts shunt require further clarification.

Despite these other potential temporizing measures, LTx remains the only definitive procedure and is more widely available compared to the reversed Potts shunt. In addition to potential survival benefits associated with LTx, children should be considered who are experiencing worsening functional capacity and/or poor quality of life in the setting of optimal medical and/or surgical management. The information presented in this study offers valuable insights into the clinical factors that may impact the outcomes of post-LTx. Therefore, it is crucial for clinicians who are responsible for the care of these vulnerable children to take these clinical factors into account when considering LTx. An important factor that seems to be influential is the age of the recipient. The current analysis showed recipient age of 6–11 years being associated with improved post-LTx survival. These findings align with the results reported by Hubbard et al who demonstrated that children with IPAH who underwent LTx at an older age had notably improved survival rates. [12]. Due to study design, we cannot determine the cause of the higher survival in the younger patient group, but we hypothesize that factors that may be influencing this finding include earlier referral in the disease course for this age group and/or improved adherence to post-LTx medication regimens as compared to older children. Future research is needed to explore this important question with the need for multi-center participation to be able to further study this important finding.

Compared to other diagnoses, children with PVD are younger overall and have a lower LAS for those who received a score. It is recognized that patients with PVD are at a disadvantage in the lung allocation system in the US, so it is worth considering submitting a petition for an exception to the LAS requirements to remove that disparity in children with PVD. [24].

The current study has several limitations, including the retrospective study design and lack of granularity of the database of a small cohort of patients. However, our study was multi-institutional, drawing from the largest registry of transplant patients currently available, which reduces potential biases observed in single-institution observational studies. Additionally, the limited number of children with PVD who receive LTx may have impacted the power of our analysis and possibly caused a reduction in the significance of the multivariate analysis. Hence, further investigation with a larger group of patients in this population is warranted in order to identify risk factors and obtain more precise information regarding the outcomes after LTx.

In conclusion, the current study showed that children with PVD had comparable survival rates after LTx to those with other indications. Importantly, these findings highlight the significance of considering LTx for children who have progressive PVD. Given the low number of children undergoing LTx, we think there should be a consensus document to provide better guidance for referring and selecting the high-risk pediatric population with PVD for LTx.

## Figures and Tables

**Table 1:** Baseline characteristics of pediatric lung transplant recipients with pulmonary vascular disease compared to other diagnoses

Variables*	PVD (n = 145)	Other (n = 839)	P value

***Recipient***			

Age, years, median [IQR]	7 [1–14]	13 [8–16]	**<0.001**

Age groups, years, n (%)	24 (16.6%)	79 (9.4%)	**<0.001**
<1	43 (29.7%)	71 (8.5%)	
1–5	26 (17.9%)	168 (20%)	
6–11	52 (35.8%)	521 (62.1%)	
11–17			

Sex, Female, n (%)	80 (55.2%)	493 (58.8%)	0.42

Race/ethnicity, n (%)	93 (64.1%)	636 (75.8%)	**0.003**
-White	52 (35.8%)	203 (24.2%)	
-Other (non-Black, non-Hispanic)			

Era	86 (59.3%)	507 (60.4%)	0.8
< 2010	59 (40.7%)	332 (39.6%)	
≥ 2010			

LAS (for > 12 years) *(n = 600)*	33.4 [29.5–38.7]	36.3 [33.5–42.5]	**< 0.001**

FVC% *(n = 643)*	78 [61–90.5]	40 [31–50]	**< 0.001**

FEV1% *(n = 639)*	69.5 [57.5–83]	26 [20–33]	**< 0.001**

Ventilator at Tx, n (%)	32 (22%)	151 (18%)	0.25

eGFR(ml/min/1.73m^2^)	14 (9.6%)	33 (4%)	**0.005**
< 60	113 (78%)	725 (86.4%)	
≥ 60	18 (12.4%)	81 (9.6%)	
Unknown			

Bilirubin	0.5 [0.3–0.8]	0.3 [0.2–0.5]	**< 0.01**

mPAP (mmHg)	67.5 [55.7–81.25]	28 [24–36]	**< 0.001**

O2 requirement at rest	1.5 [0–3.75]	2 [1–4]	**0.024**

HLA mismatch	15 (10.3%)	88 (10.5%)	a vs b: 0.88
0–3 (a)	83 (57.2%)	509 (60.7%)	
4–6 (b)	47 (32.4%)	242 (28.8%)	
Not reported			

**Donor**			

Age, years, median [IQR]	6 [1–14]	12 [6–17]	**< 0.001**

Sex, Female, n (%)	66 (45.5%)	412 (49.1%)	0.43

Race/Ethnicity, n (%)	88 (60.7%)	495 (59%)	0.7
White	57 (39.3%)	344 (41%)	
Non-White			

Cause of death, n (%)	41 (28.3%)	176 (21%)	0.09
Anoxia	14 (9.7%)	146 (17.4%)	
Stroke	77 (53.1%)	437 (52.1%)	
Head trauma	2 (1.4%)	10 (1.2%)	
CNS tumor	11 (7.6%)	52 (6.2%)	
Other			

* Categorical variables: count (percentage), Continuous variables: median [IQR]

PVD, Pulmonary Vascular Disease; LAS, Lung Allocation Score; FVC%, forced vital capacity percent predicted; FEV1%, forced expiratory volume in one second percent predicted; eGFR, estimated glomerular filtration rate; mPAP mean pulmonary arterial pressure (mmHg); HLA, human leukocyte antigen; CNS, central nervous system

**Table 2:** Univariable regression analysis for pediatric lung transplant recipients with pulmonary vascular disease

Variables*	N (Total n = 145)	HR	95% CI	P value

*Recipient*				

Era	59 (40.7%)	0.96	0.55–1.66	0.88
≥ 2010				

Age, years	143 (98.6%)	0.96	0.92–0.99	**0.035**

Age groups, years	24 (16.6%)	Reference	--	--
<1	42 (28.9%)	1.018	0.49–2.08	0.96
1–5	26 (17.9%)	0.4	0.166–0.98	**0.045**
6–11	51 (35.2%)	0.61	0.29–1.27	0.61
11–17				

Sex	65 (44.8%)	0.89	0.55–1.45	0.64
Male				

Race/ethnicity	92 (63.4%)	0.98	0.59–1.64	0.96
White				

LAS (for > 12 years)	83 (57.2%)	1.007	0.98–1.03	0.46

FVC	49 (33.8%)	1.001	0.98–1.023	0.91

FEV1	48 (33.1%)	0.997	0.97–1.02	0.83

HLA mismatch	82 (56.6%)	1.47	0.57–3.76	0.42
4–6				

eGFR	113 (77.9%)	0.68	0.37–1.5	0.34
>60				

Ventilator	32 (22%)	2.047	1.18–3.55	**0.01**

mPAP	70 (48.2%)	0.99	0.98–1.009	0.49

O2 requirement (at rest)	103 (71 %)	1.073	1.007–1.14	**0.029**

Bilirubin	108 (74.5%)	1.102	0.93–1.3	0.27

*Donor*				

Age, years	143 (98.6%)	0.98	0.95–1.008	0.16

Sex	78 (53.8%)	0.916	0.56–1.49	0.73
Male				

Race/Ethnicity	87 (60%)	0.65	0.39–1.07	0.089
White				

Cause of death	40 (27.6%)	Reference	--	--
Anoxia	14 (9.6%)	0.24	0.07–0.83	**0.024**
Stroke	76 (52.4%)	0.99	0.57–1.7	0.98
Head trauma	2 (1.3%)	0.51	0.068–3.87	0.52
CNS tumor	11 (7.5%)	0.32	0.093–1.14	0.07
Other				

Ischemia time	134 (92.4%)	1.86	1.12–3.11	**0.017**
>6 hours				

PVD, Pulmonary Vascular Disease; LAS, Lung Allocation Score; FVC, forced vital capacity; FEV1, forced expiratory volume in one second; HLA, human leukocyte antigen; eGFR, estimated glomerular filtration rate; mPAP mean pulmonary arterial pressure; CNS, central nervous system

**Table 3:** Multivariable regression analysis for all pediatric lung transplant recipients

	HR	95% CI		
		Lower	Upper	P value

**PVD vs other**	0.877	0.637	1.209	0.42

**Recipient Age**	Reference	--	--	--
**< 1**	1.25	0.768	2.037	0.37
**1–5**	0.893	0.537	1.486	0.66
**6–11**	1.543	0.971	2.451	0.06
**11–17**				

**Ventilator**	1.102	0.755	1.609	0.6

**O2 Requirement at Rest**	1.002	0.976	1.029	0.8

**Ischemic Time (hours)**	1.034	0.965	1.107	0.3

**Donor cause of death**	0.901	0.689	1.179	0.4
**Stroke vs other**				

PVD, Pulmonary Vascular Disease
